# Suicide Soon After Generalized Tonic-Clonic Seizure. A Possible Peri-Ictal Phenomenon?

**DOI:** 10.1100/tsw.2006.72

**Published:** 2006-03-20

**Authors:** Maurizio Pompili, Paolo Girardi, David Lester, Roberto Tatarelli

**Affiliations:** ^1^ Department of Psychiatry, Sant'Andrea Hospital, University of Rome “La Sapienza”, Italy; ^2^ McLean Hospital, Harvard Medical School, Belmont, MA, USA; ^3^ The Richard Stockton College of New Jersey, Pomona, USA

**Keywords:** suicide, seizure, epilepsy

## Abstract

We report the case of 36-year-old woman who had suffered from focal-unilateral right febrile grand mal since she was 9 months old as a result of neonatal asphyxia. Over the years, a modification of the clinical picture occurred and eventually the patient suffered from paranoid delusions that impaired her ability to work. After many years free from seizures, she experienced one day a generalized tonic-clonic seizure that required hospitalization and, soon thereafter, she committed suicide.

## INTRODUCTION

Suicide is a major cause of death among patients with epilepsy. Harris and Barraclough[1] reviewed 12 papers dealing with suicide and epilepsy and, by combining the data, estimated a suicide risk 5 times greater than in the general population. However, the suicide rate for the different types of epilepsy varied greatly.

Recently, Pompili et al.[2] performed a meta-analysis on 29 samples from the early 1960s to the present and confirmed that, overall, suicide among patients with epilepsy is a more frequent phenomenon than in the general population. According to Mendez and Doss[3], factors that can affect the rate of suicide among epileptic patients include: psychological stressors associated with the epilepsy, seizure type and frequency, psychic auras, and the presence of psychopathology.

We report here the case of a woman with long-term epilepsy and recent onset of paranoid delusions who committed suicide soon after a generalized tonic-clonic seizure. We were asked to counsel her significant others and to ascertain whether her suicide could have been prevented.

## CASE REPORT

Ms. R., a woman aged 36, experienced her first focal-unilateral right febrile *grand mal* when she was 9 months old. She experienced seizures again when she was 1 year old and 4 years old. She was diagnosed as having temporal lobe epilepsy. Over the years, the patient developed complex partial seizures that later became partial seizures with secondary generalization. The first physicians who treated her concluded that the symptoms were related to birth complications, specifically neonatal asphyxia. Despite the chronic neurological disorder, she developed normally and had various, although brief, work experiences, mainly as a housekeeper.

At the age of 23, the patient was taking carbamazepine (600 mg/day) and primidone (650 mg/day). She developed peripheral neuropatia caused by dintoin and was hospitalized. At the age of 26, the patient developed cyclic idiopathic edema that required further hospitalization, probably caused in part by the carbamazepine. She was put on a strict regimen to avoid further worsening of the disease and diagnosed with hypothyroidism, which had been responsible for the serious edema that she had developed. Magnetic resonance and electromyography were performed when she was hospitalized. The former did not indicate any abnormality in the brain, whereas the latter gave indication of possible polyneuropathia. During her lifetime, she never abused any drug or alcohol, but she was a heavy smoker. No head injury was ascertained in her clinical history.

As time passed, the seizures became sporadic, and the patient adjusted to her disability and experienced a period free of any neurological disability. During the last 2 years of her life, she became aware that she could work and she was ultimately employed in a college where she had to clean the offices of the administrative staff. Although she was able to work proficiently at first, after a few months she developed delusions of persecution, being sure that there were hidden microphones in her home. She felt that people were spying on her while she was working and that important secrets were stored in the offices. Her ability to work was, therefore, seriously impaired. She was asked to undergo a medical consultation where she was judged to be unable to work, and her employment was terminated.

Soon after this (during August), the patient developed negative symptoms such as autism, affective flattening, avolition, and social withdrawal. At this time, she also began to experience paranoid symptoms. DSM-IV-TR diagnosis was schizophreniform disorder as psychotic symptoms had persisted for less than 6 months. According to her father, she may have changed her daily administration of medications. She began to smoke heavily and rejected further medical consultations. It is noteworthy that, at this point in time, she had been free from documented seizures for almost 15 years.

One day, after a few months of unemployment, she had a serious generalized tonic-clonic seizure that required immediate hospitalization in the emergency department. She was administered diazepam by vein and the seizure was controlled. She refused any further medical contact, threatened legal action, and became critical of the medical staff. A few hours later, she asked to be discharged against medical approval.

The patient left the hospital and returned home where she quarreled bitterly with her father who was worried about a serious relapse. According to her father, a few minutes later she became quiet and showed affection and gratitude towards him. Together they discussed how to seek medical help for monitoring her epilepsy. Her father reported that she seemed to be in good spirits and showed no signs of psychosis. She agreed to meet a neurologist and a psychiatrist, and she sounded relieved.

She told her father that, since she was feeling fine, she was going to meet a friend upstairs. Instead, she climbed to the top of the building and committed suicide by jumping.

At that time she was taking Levetiracetam (500 mg), Lamotrigine (300 mg), and Primidone (750 mg). She had been told that Levetiracetam might increase the risk of suicide, but apparently no action was taken to investigate the emergence of suicide ideation. Over the last few months of her life, she also took haloperidol for her psychotic symptoms.

## DISCUSSION

Contrary to the classical presentation of psychiatric symptoms, our patient showed the onset of psychosis during a rather long period free of seizures[4,5,6]. Although there is no consistently reported relationship between the timing of seizures and suicidal behavior, there appears to be a significantly increased risk of suicidal behavior during postictal psychosis as compared with interictal psychosis[7]. Nevertheless, reports of completed and attempted suicide during seizure flurries show that suicide may sometimes be a fatal consequence of the peri-ictal state[3,7].

Suicide is most commonly associated with depressive disorders in the general population, but seems to be associated primarily with psychotic disorders rather than depression in epilepsy[3,7]. Janz[8] reported that suicide does not occur among patients with ongoing severe epilepsy, but rather in those patients who have just become free from seizures. According to Blumer et al.[9], suicide among patients with epilepsy is not the result of psychosocial difficulties caused by having seizures, but occurs in the presence of significant interictal and at times postictal psychopathology. In 1956, Williams[10] reported 21 such cases among his 100 patients with ictal emotional experience, and 5 of the 21 patients had made suicide attempts during the postictal phase. Mendez and Doss[3] described 4 cases of suicide in epileptic patients, 1 of whom had paranoid delusions was a postictal suicide.

According to the literature, the emergence of psychosis is traceable during ictal, postictal, and interictal states. It is possible that psychiatric disorders in epilepsy may result from the inhibitory activity that develops in reaction to excessive excitatory activity of the chronic seizure disorder[11,12]. This mechanism, which is still insufficiently understood, has been named “forced normalization” in cases where the electroencephalogram is normal, or “alternating psychosis” in cases where the clinical seizures have been suppressed. Psychiatric symptoms would be, therefore, the result of an enhanced inhibitory response, most commonly in the prodromal and postictal phases, and rarely during increased seizure activity as in peri-ictal psychosis.

Blumer et al.[9] suggested that, if predominance of inhibition results in the persistent suppression of seizures in chronic epilepsy, some patients are at risk of developing the most severe psychiatric complications. Following this model, a dysphoric disorder with suicidal depressive moods, psychosis, or a combination of both may result. Likewise, the acute engagement of inhibitory mechanisms by the seizure event tends to result in peri-ictal dysphoric symptoms that may include postictal depressive mood with suicidality[9].

One important implication emerging from the study by Blumer et al.[9] was that suppression of seizures in long-standing epilepsy may be associated with suicide risk. In their study, the five suicides registered during the 12-year period occurred in patients with long-standing partial seizures and dysphoric disorder, and occurred a short time after full control of the seizures was achieved. Suicide risk in patients with epilepsy seems associated primarily with the often sudden episodes of intense depressive mood during the interictal phase. Blumer et al.[9] reported that a high suicidal risk has been observed in patients who experience an ictal depressive mood that extends into the postictal phase for a period of 1 h to 3 days. This fact is consistent with Kraepelin’s[14] observation of dysphoric episodes in suicidal epileptic patients.

In our patient, suicide occurred just a few hours after a generalized tonic-clonic seizure, when apparently she was not depressed nor psychotic. It is hard to tell if she had thought about committing suicide before this last seizure. However, she had never attempted suicide in the past. The clinical picture over the last 2 years of her life showed that a number of psychological and interpersonal risk factors for suicide could be recognized. Apart from depression and paranoid psychosis, she was socially isolated, unemployed, and had lost faith in the treatment. Also, it would appear that the medications were not taken regularly.

What is noteworthy is that she committed suicide impulsively soon after a generalized tonic-clonic seizure. Such a relapse after many years free from seizures must have been seen as a further serious impairment to her already poor adjustment.

Of some importance is her attitude after discharge; she was relaxed and not agitated before committing suicide. This is also sometimes characteristic of schizophrenia patients who commit suicide[15], as if they had resolved their ambivalence. Suicidal individuals often visit clinicians with no apparent warning signs of imminent suicidal intent, which is consistent with this hypothesis.

Stressful life events may place individuals at an increased risk for suicidal behavior[16]. Jones et al.[17] suggested that care should be taken to assess an individual's perception of health issues and its impact on their thoughts about the future and feelings of hopelessness. We believe that in our patient, experiencing a seizure after 15 years free of seizures must have had a great impact on her hopes for the future. She had experienced many difficulties in finding a job because of her epilepsy. When she finally managed to find employment, she had been fired both because of epilepsy and paranoid delusions. She never recovered from this event. She was probably suicidal before the seizure, but the experience of the hospitalization and the need to undergo further medical examination was an additional stressor.

Clinicians should always consider the stigma attached to epilepsy that affects the patients' view of themselves and the views of other people towards them. This stigma can cause mood changes that contribute to reduced compliance to the medical regimen and to subsequent suicide. Clearly, more knowledge is needed in this field and future studies should focus on the analysis of those circumstances that lead to increased suicidality among patients with epilepsy.

## ACKNOWLEDGMENT

Supported in part by an unrestricted research grant by Eli-Lilly Italy (to MP).

## BIOSKETCHES

**Maurizio Pompili, M.D.,** psychiatrist and psychotherapist at the Dept. of Psychiatry, Sant'Andrea Hospital, University of Rome "La Sapienza". Research associate in psychiatry at McLean Hospital–Harvard Medical School, USA. He is a dedicated researcher in the field of suicidology and author of more than 100 scientific publications. He has published the first comprehensive meta-analysis of literature on suicide among epileptic patients.

**David Lester, Ph.D.,** is Professor of Psychology at the Richard Stockton College of New Jersey. He has doctoral degrees from Brandeis University (in psychology) and Cambridge University (in social and political science). He is Past-President of the International Association for Suicide Prevention.

**Paolo Girardi, M.D.,** Associate professor of psychiatry at the University of Rome "La Sapienza". Vice-Director of the residency training in psychiatry; he is in charge of the psychiatric ward at the Sant'Andrea Hospital in Rome. He is in charge of many research projects involving psychopharmacology and clinical trials.

**Roberto Tatarelli, M.D.,** Professor of Psychiatry, head of the Department of Psychiatry and director of the residency training in psychiatry at the 2nd Medical School, "La Sapienza" University, Rome; director of the psychiatry unit at the Sant'Andrea Hospital. President of the Italian Antistigma Association.
